# A single centre prospective study of three device-assisted therapies for Parkinson’s disease

**DOI:** 10.1038/s41531-023-00525-w

**Published:** 2023-06-29

**Authors:** Hugo Morales-Briceño, Ainhi D. Ha, Han-Lin Chiang, Yicheng Tai, Florence C. F. Chang, David S. Tsui, Jane Griffith, Donna Galea, Samuel D. Kim, Belinda Cruse, Neil Mahant, Victor S. C. Fung

**Affiliations:** 1 Movement Disorders Unit, Neurology Department, Westmead Hospital, Westmead, NSW 2145 Australia; 2 Sydney Medical School, University of Sydney, Sydney, NSW 2145 Australia; 3 Department of Neurology, Neurological Institute, Taipei Veterans General Hospital, Taipei City, Taiwan; 4 Department of Neurology, E-DA Hospital/I-Shou University, No.1, Yida Rd., Yanchao Dist., Kaohsiung City, 824 Taiwan

**Keywords:** Parkinson's disease, Parkinson's disease

## Abstract

Comparative studies assessing outcomes with the three device-assisted therapies could help to individualise treatment for patients living with Parkinson’s disease. We designed a single-centre non-randomised prospective observational study assessing the quality of life (QoL), motor and non-motor outcomes at 6 and 12-months in patients treated with subcutaneous apomorphine continuous 16-hours infusion (APO), levodopa-carbidopa intestinal gel (LCIG) or subthalamic nucleus deep brain stimulation (STN-DBS). In this study, 66 patients were included (13 APO; 19 LCIG; 34 STN-DBS). At baseline, cognitive, non-motor and motor scores were significantly less severe in the STN-DBS group, whereas the LCIG group had a longer disease duration and higher non-motor scores. In the APO group, there were no statistically significant changes in non-motor, motor and QoL scales. The LCIG group had significant changes in QoL and motor scales that were significant after multiple comparison analysis at 6 and 12-months. The STN-DBS group showed improvement in QoL scores and non-motor and motor scores at 6 and 12-months after multiple comparison analysis. In this real-life prospective study, device-assisted therapies showed differences in their effects on QoL and motor and non-motor function at 12-months. However, there were also differences in baseline characteristics of the patient groups that were not based on pre-determined selection criteria. Differences in characteristics of patients offered and/or treatment with different device-assisted therapies may reflect within-centre biases that may, in turn, influence perceptions of treatment efficacy or outcomes. Treatment centres should be aware of this potential confounder when assessing and offering device-assisted treatment options to their patients and potential baseline differences need to be taken into consideration when comparing the results of non-randomised studies.

## Introduction

The emergence of motor and non-motor fluctuations causes substantial changes in the quality of life and functional independence of people with Parkinson’s disease (PD). Modification of oral dopaminergic therapy provides adequate improvement in only a subset of patients. Limitations of oral therapies include delayed or erratic absorption of levodopa, pulsatile dopaminergic stimulation and inherent disease progression^1–4^. Device-assisted therapies (DATs) are specific treatment modalities for PD patients that include 16-h continuous subcutaneous apomorphine (APO), 16-h levodopa-carbidopa intestinal gel (LCIG), and bilateral globus pallidus pars interna (GPi) or subthalamic nucleus (STN) deep brain stimulation (DBS). Several RCTs and observational studies have provided evidence of the benefits in motor and non-motor symptoms and quality of life (QoL) with treatment with any of these options^1,5–7^.

Current expert recommendations have stressed the importance of the number of levodopa doses per day (≥5 doses), time spent with troublesome dyskinesia (≥1 h/day) and “off” symptoms (≥2 h) and lack of significant dementia as important indications for DATs^8^. Other considerations include patient preference, treatment availability, cost of treatment and local expertise. Furthermore, evidence suggests that different phenotypes or subtypes of PD are associated with different risk of developing non-motor symptoms such as dementia. For example, those with troublesome REM-sleep behaviour disorders and greater olfactory dysfunction. Therefore, PD phenotypes may influence potential responses to different DATs^9,10^.

Despite a wealth of studies on the efficacy of each device-assisted therapy, there are few studies which compare outcomes between therapies, and only one study has compared the three therapies^11,12^. Here we report the results of a single-centre, prospective, non-randomised real-life observational study that evaluated clinical, functional and QoL outcomes at 6 and 12-months in patients with APO, LCIG and STN-DBS therapies.

## Results

**Fig. 1:**
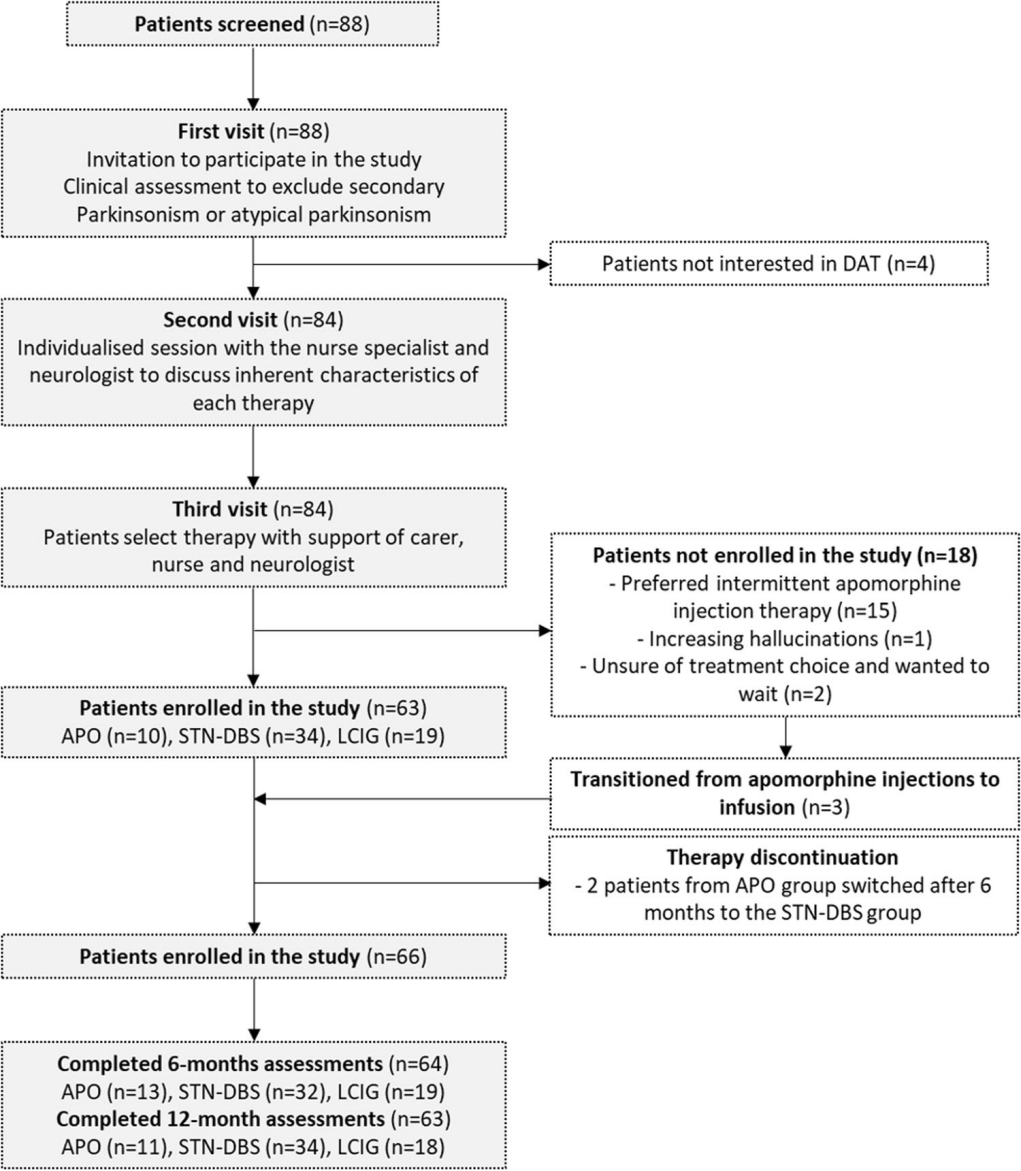
The figure illustrates the process of patient selection before the commencement of device-assisted therapy. All patients included in the study were seen at least twice in our Movement Disorders Clinic before study enrolment. Treatment was decided between the patient and treating clinician according to routine clinical care, based on factors such as patient preference and suitability for each of the treatment options in the opinion of the treating movement disorder specialist. A more detailed description of patient selection is provided in the text. DAT device-assisted therapy, APO apomorphine, LCIG levodopa-carbidopa intestinal gel infusion, STN-DBS subthalamic nucleus deep brain stimulation.

A total of 88 patients were screened from 2013 to 2019. After the subsequent assessments before treatment commencement, 18 patients did not enter the study (see Fig. 1). Fifteen of these patients commenced intermittent apomorphine injections. After six months of apomorphine subcutaneous injections, three of these patients were started on APO infusion and completed 6 and 12-month examinations, thus, they were included in the final analysis.

**Fig. 2:**
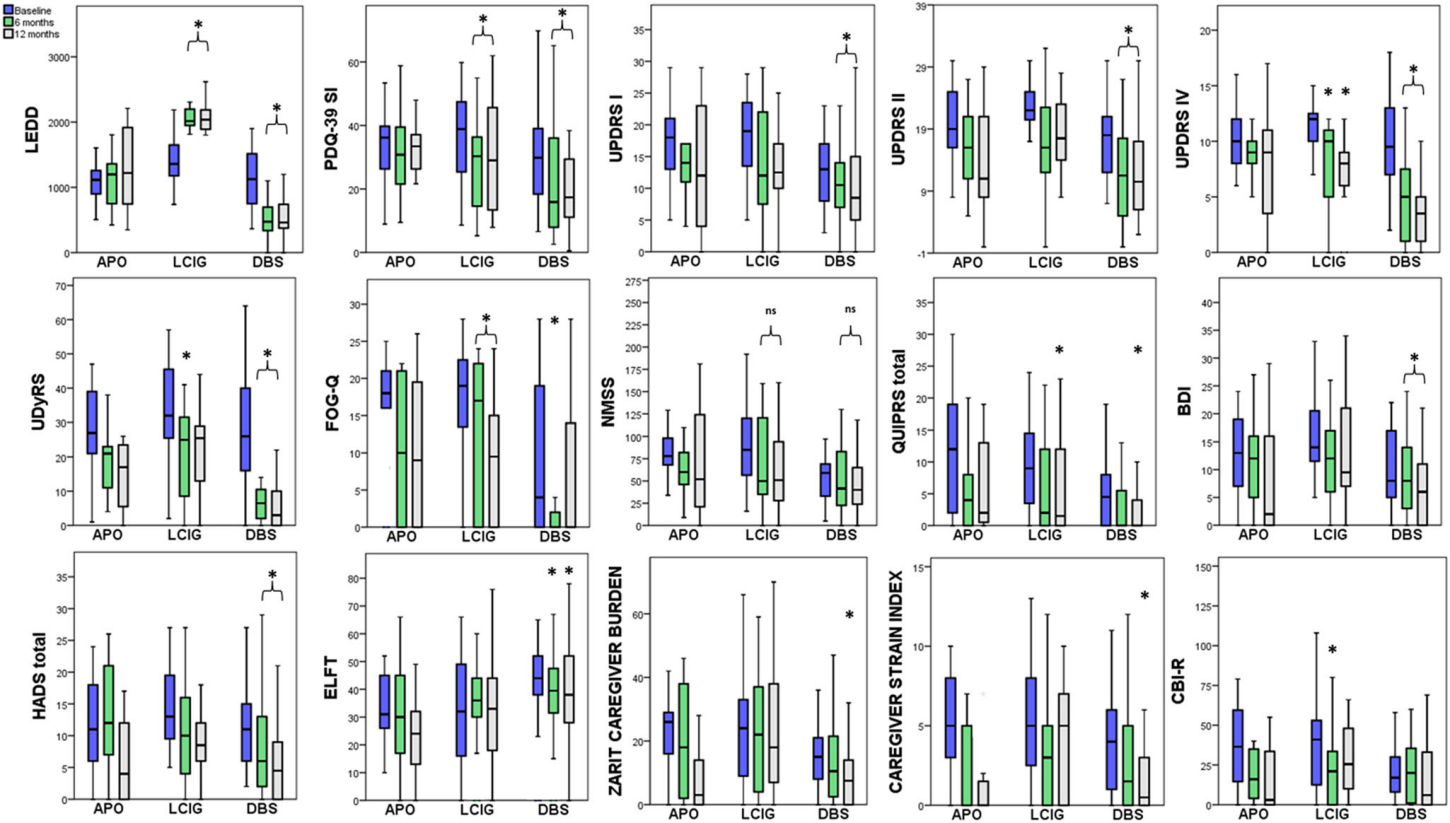
The boxplots show the median and interquartile values of clinimetric assessments at baseline (blue box), 6 (green box) and 12 months (light grey box) in the APO, LCIG and DBS treatment groups. The boxplots show the results of the statistical analysis using the Kruskal–Wallis method. The asterixis represents significant *p* values after Bonferroni correction (<0.05). The centre line within the box represents the median value, whereas the upper and lower interquartile ranges are represented in the upper and lower sections from the median line within the box. The whiskers represent the maximum (from the top of the box) and minimum values (from the lower part of the box), respectively. Curly brackets with asterixis are used when statistical significance was reached at 6 and 12-months. Outliers are not shown. APO apomorphine, LCIG levodopa-carbidopa intestinal gel infusion, DBS subthalamic nucleus deep brain stimulation, LEDD levodopa equivalent daily dose, PDQ 39 39 item Parkinson’s Disease Questionnaire SI, UPDRS-I, UPDRS-II, UPDRS-IV refers to the movement disorder society unified Parkinson’s disease rating scale subscores Part I (Non-motor aspects of experiences of daily living), Part II (Motor aspects of experiences of daily living) and Part III (Motor examination) respectively, UDysRS unified dyskinesia rating scale; FOG-Q new freezing of gait questionnaire, NMSS non-motor symptoms scale, QUIP-RS questionnaire for impulsive-compulsive control disorders in Parkinson’s disease, BDI Beck depression inventory, HADS Hospital anxiety and depression scale, ELF excluded letter fluency, ZCB Zarit caregiver burden scale, CSI caregiver strain index, CBI-R Cambridge behavioural inventory revised.

In total, 66 patients (46 males and 20 females) were enrolled and had treatment with DATs (13 APO; 19 LCIG; 34 STN-DBS). See Table 1 for the baseline characteristics of each group. Two patients with APO discontinued therapy at 6-months due to the lack of adequate control of motor fluctuations. These two patients were transitioned to STN-DBS and completed 6 and 12-months assessments, thus are part of the STN-DBS group analysis. Two patients in the STN-DBS missed the 6-month assessments but had the 12-month assessments. One patient in the LCIG group missed his 12-month assessments due to intercurrent illness. Outcomes at 6 and 12-months and the number completing these assessments are summarised in Tables 2 and 3. Figure 2 shows the median and IQR scores in motor, non-motor and caregiver scales from baseline to 6 and 12-months for each treatment group.

**Table 1:** Baseline clinical characteristics and assessments of the study cohort.

	Total	APO	LCIG	STN-DBS	*P* value
Number of subjects	66	13	19	34	
Age of PD	51.0 (45.0–57.0)	51.0 (47.2–57.6)	52.0 (44.0–57.0)	51.0 (44.7–56.0)	0.859
Age at intervention	62.0 (57.0–69.0)	60.5 (54.7–68.5)	67.0 (59.0–73.0)	62.5 (55.0–67.2)	0.130
PD duration (years)	10.0 (8.0–13.0)	10.0 (8.0–11.0)	13.0 (10.0–18.0)*	10.0 (7.0–13.0)	**0.002**
LEDD (mg)	1214.0 (850–1570.0)	1098.0 (859.0–1289.7)	1358.0 (1175.0–1696.0)	1125.7 (741.2–1520.5)	0.240
**Quality of life**					
PDQ-39 SI	34.0 (20.3–40.7)	34.8 (23.5–39.7)	38.8 (23.8–49.2)	29.8 (17.7–39.4)	0.131
**Non-motor symptoms**					
UPDRS-I	15.0 (11.0–21.0)	16.5 (12.5–21.0)	19.0 (13.0–24.0)	13.0 (8.0–17.5)**	**0.031**
NMSS	68.0 (43.0–88.0)	66.0 (43.5–98.0)	85.0 (53.0–121.0)*	59.0 (31.5–71.2)	**0.005**
BDI	12.0 (6.0–18.0)	13.0 (6.5–19.2)	14.0 (11.0–23.0)	8.0 (4.7–17.0)	0.052
HADS	11.0 (7.0–15.0)	10.5 (6.0–18.2)	13.0 (9.0–20.0)	11.0 (5.7–15.0)	0.277
HADS-A	6.0 (3.0–9.0)	6.0 (3.0–8.2)	8.0 (4.0–11.0)	5.5 (3.0–7.2)	0.254
HADS-D	5.0 (3.0–8.0)	4.5 (2.0–10.0)	6.0 (4.0–9.0)	5.0 (3.0–7.2)	0.241
QUIP-RS	6.0 (1.0–14.0)	11.0 (2.0–19.0)	9.0 (2.0–15.0)	4.5 (0.0–8.0)***	**0.040**
MoCA	27.0 (25.0–29.0)	26.0 (25.0–27.5)	26.0 (24.0–28.0)	28.0 (25.0–30.0)	0.189
ELFT	41.0 (26.0–51.0)	31.5 (25.5–45.7)	32.0 (13.0–51.0)	44.0 (37.0–52.2)	0.082
ACE-III	91.0 (85.0–94.0)	86.0 (77.2–90.0)	87.0 (81.0–93.0)	91.5 (88.5–95.0)***	**0.001**
**Motor function**					
UPDRS-II	20.0 (15.0–24.0)	20.0 (16.0–25.0)	22.0 (20.0–25.0)	18.0 (12.0–21.5)**	**0.041**
UPDRS-III “Off-state”	46.0 (38.0–57.0)	47.0 (42.5–65.7)	50.0 (42.0–64.0)	40.0 (34.0–55.0)***	**0.048**
UPDRS-III “On-state”	28.0 (20.0–32.0)	29.5 (25.2–36.0)	30.0 (24.0–36.0)	23.5 (17.0–30.0)***	**0.032**
UPDRS-IV	10.0 (8.0–12.0)	10.0 (7.7–12.0)	12.0 (10.0–13.0)	9.5 (7.0–13.0)	0.342
HY	1.0 (1.0–2.0)	1.5 (1.0–2.0)	2.0 (1.0–3.0)	1.0 (1.0–2.0)	0.144
FOG-Q	16.0 (0.0–21.0)	18.5 (14.0–21.0)	19.0 (13.0–23.0)	4.0 (0.0–19.2)**	**0.006**
SEADL	80.0 (70.0–90.0)	80.0 (70.0–82.5)	70.0 (60.0–80.0)^&^	80.0 (80.0–90.0)	**0.049**
UDysRS	28.0 (20.0–41.0)	31.5 (19.7–40.0)	32.0 (25.0–48.0)	26.0 (14.7–40.7)	0.283
**Caregiver scales**					
ZCB	17.0 (8.0–29.0)	25.5 (11.5–29.2)	24.0 (7.0–36.0)	15.0 (7.7–21.0)	0.185
CSI	4.0 (2.0–7.0)	5.0 (1.7–8.0)	5.0 (2.0–8.0)	4.0 (0.7–6.2)	0.322
CBI-R	27.0 (9.0–43.5)	35.0 (14.5–35.0)	41.0 (10.0–53.0)	17.0 (7.0–30.0)***	**0.026**

All data were expressed as median with IQR. The asterisks and & symbol represent the group that had different baseline characteristics from the other two groups after Kruskal–Wallis analysis. Significant *p* value (<0.05) are shown in bold.

*APO* apomorphine, *STN-DBS* subthalamic nucleus deep brain stimulation, *LCIG* levodopa-carbidopa intestinal gel infusion, *PD* Parkinson’s disease, *H&Y* Hoehn & Yahr scale, *LEDD* levodopa equivalent daily dose, *MDS-UPDRS* Movement Disorder Society Unified Parkinson’s disease rating scale, *UDysRS* unified dyskinesia rating scale, *PDQ* 39 39 item Parkinson’s Disease Questionnaire SI, *SEADL* Schwab and England activities of daily living scale, *FOG-Q* new freezing of gait questionnaire, *NMSS* non-motor symptoms scale, *BDI* Beck depression inventory, *HADS* hospital anxiety and depression scale, *HADS-A* hospital anxiety and depression scale anxiety score, *HADS-D* hospital anxiety and depression scale depression score, *QUIP-RS* questionnaire for impulsive-compulsive control disorders in Parkinson’s disease, *MOCA* Montreal cognitive assessment, *ELF* excluded letter fluency, *ACE-III* Addenbrooke’s cognitive examination III, *CSI* caregiver strain index, *CBI-R* Cambridge behavioural inventory revised, *ZCB*- Zarit caregiver burden scale.

*Statistically significant when compared to APO and STN-DBS; ** Statistically significant when compared to LCIG; *** Statistically significant when compared to APO and LCIG and statistically significant when compared to STN-DBS.

^&^Symbol represent the group that had different baseline characteristics from the other two groups after Kruskal–Wallis analysis.

**Table 2:** Motor and non-motor outcomes at 6 months within treatment arms.

		APO				LCIG					STN-DBS			
	*n*	Baseline	6-months	Friedman	*n*	Baseline	6-months	Friedman	Kruskal–Wallis	*n*	Baseline	6-months	Friedman	Kruskal–Wallis
**LEDD (mg)**	13	1098.0 (859.0–1289.7)	1190.0 (722.5–1390.1)	0.519	19	1358.0 (1175.0–1696.0)	2014.5 (1928.8–2223.7)	<**0.001**	<0.001*	32	1125.7 (741.2–1520.5)	475.0 (300.0–720.0)	< **0.001**	<0.001*
**Quality of life**														
PDQ-39 SI	11	34.8 (23.5–39.7)	29.9 (21.5–39.4)	0.717	19	38.8 (23.8–49.2)	30.3 (11.9–37.5)	**0.022**	0.013*	32	29.8 (17.7–39.4)	15.8 (7.5–40.0)	**0.019**	0.018*
**Non-motor symptoms**														
UPDRS-I	11	16.5 (12.5–21.0)	14.5 (10.5–17.0)	0.558	19	19.0 (13.0–24.0)	12.0 (7.0–23.0)	0.267		32	13.0 (8.0–17.5)	10.5 (7.0–14.0)	**<0.001**	<0.001*
NMSS	11	78.5 (65.7–100.5)	66.0 (43.5–98.0)	0.779	19	85.0 (53.0–121.0)	50.0 (32.0–128.0)	0.333		32	59.0 (31.5–71.2)	41.5 (22.2–83.5)	0.189	
BDI	11	13.0 (6.5–19.2)	11.0 (4.2–16.0)	0.441	19	14.0 (11.0–23.0)	12.0 (5.0–18.0)	0.205		32	8.0 (4.7–17.0)	8.0 (3.0–14.0)	**0.001**	0.033*
HADS	11	10.5 (6.0–18.2)	11.0 (6.0–21.7)	0.109	19	13.0 (9.0–20.0)	10.0 (3.0–16.0)	0.058		32	11.0 (5.7–15.0)	6.0 (2.0–13.0)	**0.001**	0.005*
HADS-A	11	6.0 (3.0–8.2)	6.0 (3.5–11.7)	0.172	19	8.0 (4.0–11.0)	5.0 (2.0–9.0)	**0.043**	0.405	32	5.5 (3.0–7.2)	3.5 (1.0–7.0)	**<0.001**	0.012*
HADS-D	11	4.5 (2.0–10.0)	5.5 (1.7–7.2)	0.233	19	6.0 (4.0–9.0)	4.0 (1.0–9.0)	0.091		32	5.0 (3.0–7.2)	3.0 (1.2–6.0)	**0.027**	0.016*
QUIP-RS	11	11.0 (2.0–19.0)	4.0 (0.0–9.2)	0.337	19	9.0 (2.0–15.0)	2.0 (0.0–13.0)	**0.015**	**0.037**	32	4.5 (0.0–8.0)	0.0 (0.0–6.5)	**0.007**	**0.046**
MoCA	11	26.0 (25.0–27.5)	26.5 (21.7–28.2)	0.862	19	26.0 (24.0–28.0)	26.0 (21.0–28.0)	0.594		32	28.0 (25.0–30.0)	27.0 (24.2–29.0)	0.580	
ELFT	11	31.5 (25.5–45.7)	31.0 (16.2–45.7)	0.338	19	32.0 (13.0–51.0)	36.0 (30.0–45.0)	0.338		32	44.0 (37.0–52.2)	39.5 (31.2–47.7)	**0.001**	0.010*
ACE-III	11	86.0 (77.2–90.0)	90.0 (81.0–94.2)	0.915	19	87.0 (81.0–93.0)	86.0 (76.0–96.0)	0.678		32	91.5 (88.5–95.0)	92.5 (89.0–96.0)	0.056	
**Motor function**														
UPDRS-II	11	20.0 (16.0–25.0)	16.5 (11.0–21.0)	0.203	19	22.0 (20.0–25.0)	16.0 (12.0–25.0)	0.059		32	18.0 (12.0–21.5)	11.5 (5.0–18.5)	**0.001**	0.003*
UPDRS-III “On”	13	29.5 (25.2–36.0)	25.5 (17.5–36.0)	0.205	19	30.0 (24.0–36.0)	25.0 (13.0–37.0)	0.744		32	23.5 (17.0–30.0)	17.0 (12.5–28.0)	0.090	
UPDRS-IV	13	10.0 (7.7–12.0)	9.0 (7.7–12.0)	0.226	19	12.0 (10.0–13.0)	10.0 (5.0–11.0)	**0.003**	0.011*	32	9.5 (7.0–13.0)	5.0 (1.0–7.7)	**<0.001**	<0.001*
H&Y	13	1.5 (1.0–2.0)	2.0 (1.0–3.0)	0.256	19	2.0 (1.0–3.0)	2.0 (1.0–2.0)	0.905		32	1.0 (1.0–2.0)	2.0 (1.0–2.0)	**0.019**	0.118
FOG-Q	11	18.5 (14.0–21.0)	13.5 (0.0–21.2)	0.976	19	19.0 (13.0–23.0)	17.0 (0.0–22.0)	**0.004**	0.010*	32	4.0 (0.0–19.2)	0.0 (0.0–3.0)	**0.015**	**0.034**
SEADL	11	80.0 (70.0–82.5)	80.0 (77.5–82.5)	0.697	19	70.0 (60.0–80.0)	80.0 (70.0–90.0)	0.087		32	80.0 (80.0–90.0)	90.0 (80.0–97.5)	0.124	
UDysRS	11	31.5 (19.7–40.0)	21.5 (10.2–25.0)	0.307	19	32.0 (25.0–48.0)	25.0 (8.0–33.0)	**0.026**	0.016*	32	26.0 (14.7–40.7)	6.5 (2.0–10.7)	**<0.001**	<0.001*
**Caregiver scales**														
ZCB	11	25.5 (11.5–29.2)	17.5 (1.5–39.5)	0.353	19	24.0 (7.0–36.0)	22.0 (4.0–39.0)	0.831		32	15.0 (7.7–21.0)	10.5 (2.2–22.2)	**0.011**	0.104
CSI	11	5.0 (1.7–8.0)	3.5 (0.0–5.5)	0.142	19	5.0 (2.0–8.0)	3.0 (0.0–6.0)	0.399		32	4.0 (0.7–6.2)	1.5 (0.0–5.0)	**0.003**	**0.046**
CBI-R	11	35.0 (14.5–35.0)	16.0 (6.0–36.2)	0.249	19	41.0 (10.0–53.0)	21.0 (0.0–35.0)	**0.030**	0.012*	32	17.0 (7.0–30.0)	20.0 (1.0–36.7)	0.233	

All data were expressed as median ± interquartile range. The *p* values of Friedman analysis are shown in the third column of each treatment group. If the *p* value was significant (bold), then a second comparison analysis using Kruskal–Wallis is shown in the next column. If the resulted *P* value was significant (bold) and survived Bonferroni correction, an asterixis is shown next to the *p* value.

*APO* apomorphine, *STN-DBS* subthalamic nucleus deep brain stimulation, *LCIG* levodopa-carbidopa intestinal gel infusion, *PD* Parkinson’s disease, *H&Y* Hoehn & Yahr scale, *LEDD* levodopa equivalent daily dose, *MDS-UPDRS* movement disorder society unified Parkinson’s disease rating scale, *UDysRS* unified dyskinesia rating scale, *PDQ 39* 39 item Parkinson’s disease questionnaire SI, *SEADL* Schwab and England activities of daily living scale, *FOG-Q* new freezing of gait questionnaire, *NMSS* non-motor symptoms scale, *BDI* Beck depression inventory, *HADS* hospital anxiety and depression scale, *HADS-A* hospital anxiety and depression scale anxiety score, *HADS-D* hospital anxiety and depression scale depression score, *QUIP* questionnaire for impulsive-compulsive control disorders in Parkinson’s disease, *MOCA* Montreal cognitive assessment, *ELF* excluded letter fluency, *ACE-III* Addenbrooke’s cognitive examination III, *CSI* caregiver strain index, *CBI-R* Cambridge behavioural inventory revised, *ZCB* Zarit caregiver burden scale.

**Table 3:** Motor and non-motor outcomes at 12 months within treatment arms.

		APO				LCIG					STN-DBS			
	*n*	Baseline	12-months	Friedman	*n*	Baseline	12-months	Friedman	Kruskal–Wallis	*n*	Baseline	12-months	Friedman	Kruskal–Wallis
**LEDD (mg)**	11	1098.0 (859.0–1289.7)	12193 (692.5–1979.0)	0.519	18	1358.0 (1175.0–1696.0)	2038.0 (1871.2–2190.0)	<**0.001**	<0.001*	34	1125.7 (741.2–1520.5)	462.5 (368.7–742.5)	< **0.001**	<0.001*
**Quality of life**														
PDQ-39 SI	9	34.8 (23.5–39.7)	33.4 (23.9–42.5)	0.717	17	38.8 (23.8–49.2)	29.0 (13.1–45.6)	**0.022**	0.022*	29	29.8 (17.7–39.4)	17.3 (10.5–29.6)	**0.019**	0.012*
**Non-motor symptoms**														
UPDRS-I	9	16.5 (12.5–21.0)	14.5 (3.0–25.0)	0.558	17	19.0 (13.0–24.0)	12.5 (9.7–17.2)	0.267		34	13.0 (8.0–17.5)	8.5 (5.0–15.0)	**<0.001**	<0.001*
NMSS	9	78.5 (65.7–100.5)	54.5 (19.5–134.5)	0.779	18	85.0 (53.0–121.0)	51.0 (26.7–104.2)	0.333		34	59.0 (31.5–71.2)	40.0 (22.5–66.2)	0.189	
BDI	9	13.0 (6.5–19.2)	4.5 (0.0–18.0)	0.441	17	14.0 (11.0–23.0)	95. (6.2–22.0)	0.205		26	8.0 (4.7–17.0)	6.0 (0.0–11.0)	**0.001**	<0.001*
HADS total	9	10.5 (6.0–18.2)	5.0 (0.013.0)	0.109	17	13.0 (9.0–20.0)	8.5 (5.5–12.2)	0.058		27	11.0 (5.7–15.0)	4.5 (0.0–9.0)	**0.001**	<0.001*
HADS-A	9	6.0 (3.0–8.2)	2.5 (0.0–7.2)	0.172	17	8.0 (4.0–11.0)	5.0 (2.0–7.0)	**0.043**	0.016*	27	5.5 (3.0–7.2)	5.0 (3.0–7.2)	**<0.001**	<0.001*
HADS-D	9	4.5 (2.0–10.0)	1.5 (0.0–5.7)	0.233	17	6.0 (4.0–9.0)	4.0 (2.7–6.2)	0.091		27	5.0 (3.0–7.2)	2.0 (0.0–6.0)	**0.027**	**0.049**
QUIP-RS	9	11.0 (2.0–19.0)	2.5 (0.2–14.5)	0.337	17	9.0 (2.0–15.0)	1.5 (0.0–12.5)	**0.015**	0.008*	34	4.5 (0.0–8.0)	0.0 (0.0–4.5)	**0.007**	0.012*
MoCA	9	26.0 (25.0–27.5)	25. (15.2–27.0)	0.862	17	26.0 (24.0–28.0)	24.5 (18.7–28.0)	0.594		34	28.0 (25.0–30.0)	28.0 (26.0–29.0)	0.580	
ELFT	9	31.5 (25.5–45.7)	25.5 (10.5–36.7)	0.338	15	32.0 (13.0–51.0)	33.0 (17.2–46.2)	0.338		30	44.0 (37.0–52.2)	38.0 (28.0–52.2)	**0.001**	0.001*
ACE-III	9	86.0 (77.2–90.0)	84.5 (65.7–90.7)	0.915	17	87.0 (81.0–93.0)	82.5 (70.0–94.0)	0.678		33	93.5 (90.5–96.0)	92.5 (89.0–96.0)	0.056	
**Motor function**														
UPDRS-II	9	20.0 (16.0–25.0)	13.5 (8.0–21.0)	0.203	17	22.0 (20.0–25.0)	17.5 (13.7–23.5)	0.059		34	18.0 (12.0–21.5)	10.5 (6.0–17.2)	**0.001**	<0.001*
UPDRS-III “On”	11	29.5 (25.2–36.0)	20.0 (7.5–30.2)	0.205	17	30.0 (24.0–36.0)	24.5 (15.7–42.5)	0.744		34	23.5 (17.0–30.0)	18.0 (14.5–26.0)	0.090	
UPDRS-IV	11	10.0 (7.7–12.0)	9.0 (0.0–12.0)	0.226	17	12.0 (10.0–13.0)	8.0 (5.7–9.5)	0.003	0.001*	34	9.5 (7.0–13.0)	3.5 (1.0–5.2)	**<0.001**	<0.001*
H&Y	9	1.5 (1.0–2.0)	2.0 (1.0–2.0)	0.256	17	2.0 (1.0–3.0)	1.5 (1.0–2.2)	0.905		34	1.0 (1.0–2.0)	2.0 (1.0–2.0)	**0.019**	0.104
FOG-Q	10	18.5 (14.0–21.0)	10.0 (0.0–23.5)	0.976	17	19.0 (13.0–23.0)	9.5 (0.0–16.0)	0.004	0.004*	34	4.0 (0.0–19.2)	0.0 (0.0–14.5)	**0.015**	0.492
SEADL	9	80.0 (70.0–82.5)	80.0 (30.0–90.0)	0.697	16	70.0 (60.0–80.0)	80.0 (70.0–90.0)	0.087		34	80.0 (80.0–90.0)	90.0 (80.0–100.0)	0.124	
UDysRS	10	31.5 (19.7–40.0)	17.0 (0.0–25.0)	0.307	17	32.0 (25.0–48.0)	25.5 (12.5–29.2)	0.026	**0.026**	34	26.0 (14.7–40.7)	3.0 (0.0–10.2)	**<0.001**	<0.001*
**Caregiver scales**														
ZCB	9	25.5 (11.5–29.2)	3.5 (0.0–15.0)	0.353	15	24.0 (7.0–36.0)	18.0 (6.7–38.5)	0.831		24	15.0 (7.7–21.0)	7.5 (0.0–14.2)	**0.011**	0.004*
CSI	9	5.0 (1.7–8.0)	1.0 (0.0–3.5)	0.142	15	5.0 (2.0–8.0)	5.0 (0.0–7.0)	0.399		24	4.0 (0.7–6.2)	0.5 (0.0–3.5)	**0.003**	0.004*
CBI-R	9	35.0 (14.5–35.0)	3.0 (0.0–34.7)	0.249	13	41.0 (10.0–53.0)	25.5 (8.5–49.2)	**0.030**	0.211	24	17.0 (7.0–30.0)	6.0 (0.0–33.2)	0.233	

All data were expressed as median ± interquartile range. The *p* values of Friedman analysis are shown in the third column of each treatment group. If the *p* value was significant (bold), then a second comparison analysis using Kruskal–Wallis is shown in the next column. If the resulted *P* value was significant (bold) and survived Bonferroni correction, an asterixis is shown next to the *p* value.

*APO* apomorphine, *STN-DBS* subthalamic nucleus deep brain stimulation, *LCIG* levodopa-carbidopa intestinal gel infusion, *PD* Parkinson’s disease, *H&Y* Hoehn & Yahr scale, *LEDD* levodopa equivalent daily dose, *MDS-UPDRS* movement disorder society unified Parkinson’s disease rating scale, *UDysRS* unified dyskinesia rating scale, *PDQ-3*9 39 item Parkinson’s disease questionnaire SI, *SEADL* Schwab and England activities of daily living scale, *FOG-Q* new freezing of gait questionnaire, *NMSS* non-motor symptoms scale, *BDI* Beck depression inventory, HADS hospital anxiety and depression scale, *HADS-A* hospital anxiety and depression scale anxiety score, *HADS-D* hospital anxiety and depression scale depression score, *QUIP* questionnaire for impulsive-compulsive control disorders in Parkinson’s disease, *MOCA* Montreal cognitive assessment, *ELF* excluded letter fluency, *ACE-III* Addenbrooke’s cognitive examination III, *CSI* Caregiver strain index, *CBI-R* Cambridge behavioural inventory revised, *ZCB* Zarit caregiver burden scale.

### Baseline characteristics of the cohort

The median age at intervention of the entire cohort was 62.0 years (IQR 57.0–69.0). At baseline, there were no age differences at the time of therapy initiation between groups. The comparative analysis (Kruskal–Wallis) showed that patients in the LCIG group had a longer disease duration (*P* = 0.002) and higher NMSS scores at baseline (*P* = 0.005) compared to APO and STN-DBS groups. In addition, SEADL scores were more affected in the LCIG group (*P* = 0.049) compared to the STN-DBS group. In contrast, the STN-DBS group had lower UPDRS-I scores (*P* = 0.031) when compared to the LCIG group. Moreover, QUIP-RS (*P* = 0.040), ACE-III (*P* = 0.001), UPDRS-III “on-state” (*P* = 0.032) and “off-state” (*P* = 0.048), FOG-Q (*P* = 0.006) and CBI-R (*P* = 0.026) were less severe in the STN group when compared to both APO and LCIG groups. At baseline, off-time scores (UPDRS-IV subscore 4.3; time spent in the off-state) across groups was similar. APO patients had 1.6 ± 0.5 points, the STN-DBS group had 1.5 ± 0.7 points, and the LCIG group had 1.5 ± 0.7 points. There were no differences in the remainder of baseline characteristics and clinical scales. Baseline caregiver stress scores were similar between groups.

### Infusion rates, stimulation parameters and follow-up visits

The total LEDD in the APO group remained unchanged from the baseline compared to 6 and 12-months (see Tables 2 and3). However, in the LCIG group, median (IQR) baseline total LEDD increased from 1358.0 mg (1175.0–1696.0) to 2014.5 mg (1928.8–2223.7) at 6-months (*P* = <0.001) and 2038.0 mg (1871.2–2190.0) at 12-months (*P* = <0.001) whereas, in the STN group, this was reduced from 1125.7 mg (741.2–1520.5) to 475.0 mg (300.0–720.0) at 6-months (*P* = <0.001) and 462.5 mg (368.7–742.5) at 12-months (*P* = <0.001). All these changes were significant after the Bonferroni correction.

All patients (*n* = 13) with APO commenced with a 16-h infusion. At 12-months, the mean continuous rate was 0.76 ml/h (range, 0.2–1.0 ml/h), equivalent to 3.8 mg/h (range, 1–5 mg/h) and a bolus dose of 0.42 ml (range, 0.1–0.9 ml) equivalent to 2.1 mg (range, 0.5–4.5 mg). Only one patient had a 24-h infusion at 12-months. In the LCIG group, all patients were commenced on a 16-h infusion; however, after six months, four patients were transitioned to a 24-h infusion. The indication for a round-the-clock infusion was freezing of gait (present at baseline in three, one had new onset), which was not reduced with a 16-h infusion. The average infusion rates at 12-months in all 19 patients were as follows: morning dose, mean 7.5 ml (range, 2.5–12 ml); continuous daytime rate, 4.7 ml (range, 2.6–7 ml) and extra dose, 1.8 ml (range, 0.8–3 ml). In the patients with 24-h infusion, the mean night-time rate was 2.9 ml (range, 1.6–4.9 ml). In the STN-DBS group, patients had their initial stimulation parameters with conventional settings, and at 12-months, the parameters were: R STN mean amplitude 2.7 V (range, 0.7–4.3 V), L STN mean amplitude 2.7 V (range, 1.1–4.5 V), with an average pulse width of 60 ms and frequency of 130 Hz. However, at 12-months, three patients changed their stimulation settings; two had low-frequency stimulation (70 Hz) for freezing of gait (FOG) and another to interleaving stimulation trying to ameliorate stimulation-induced upper limb dystonia (see Supplementary Table 1 for stimulation settings for each patient).

The number of clinic visits for the three different groups was different. During 12-months, the median number of clinical visits in the APO group was 10, whereas the LCIG group had 15 visits, and the STN-DBS group had 8 visits.

### Safety data

Adverse events occurred in 59% of the cohort (see Supplementary Table 1 for details). Only clinically significant adverse events identified during routine clinical follow-up or at their formal 6 and 12-month assessments are reported, and none were severe nor resulted in hospitalisation or death. In the APO group, eleven patients had at least one side effect, including impulse control disorder (ICD) in three female patients (all with excessive shopping and in co-treatment with pramipexole), excessive daytime sleepiness in two, and worsening of parkinsonism with FOG in two. Only one patient reported non-troublesome hallucinations. The patients with ICD had improvement in excessive shopping behaviour after slow titration of pramipexole. The two patients with excessive daytime sleepiness during the first weeks of APO introduction did not require a reduction in their infusion rate. Six months after starting APO infusion, two patients in whom the parkinsonism (also with FOG) was exacerbated switched to STN-DBS. In the LCIG, nine patients had side effects, including hallucinations, FOG and impulse control disorder (a female with a previous history of excessive shopping whilst during treatment with pramipexole) occurred, one in each, whereas non-troublesome dyskinesia and cognitive change were observed in two patients each.

In the STN-DBS, there were 20 patients that experienced side effects, with the main being speech problems in four and freezing of gait in three. An increase in REM-sleep behaviour disorder symptoms was reported by the carers of two patients; an observation that has been made by others in some cases after STN-DBS^13^. Three of the patients with FOG were treated by increasing the total LEDD, which reduced the FOG in all; however, two patients additionally had their stimulation settings changed to low-frequency stimulation with good effect. Three patients with well-controlled motor fluctuations developed apathy within 6-months after STN-DBS, none of which had changed in depression or anxiety scales nor had dopamine agonist withdrawal syndrome. At 12-months of these patients, apathy was reduced with the treatment of anti-depressants.

### Effect of DATs on quality of life

Tables 2, 3, show the comparative analysis between baseline and 6 and-12 months, respectively. Hereafter, we describe the *p* values from Kruskal–Wallis analysis and report if these were significant after the Bonferroni correction. PDQ-39 SI did not change at 6 and 12-months in the APO group. In the LCIG group, PDQ-39 SI improved at 6-months (*P* = 0.013) and 12-months (median IQR change from baseline: −9.9 IQR 10.7–4.0; *P* = 0.022), both significant after Bonferroni correction. In the STN-DBS group, PDQ-39 SI improved at 6-months (*P* = 0.018) and 12-months (median IQR change from baseline: −12.5 IQR 5.2–9.8; *P* = 0.012), both significant after Bonferroni correction.

### Effect of DATs on non-motor symptoms

In the APO group, there was no change in NMSS, BDI, HADS total, QUIP-RS, UPDRS-I and cognitive assessment scales.

The LCIG group did not have a reduction in NMSS scores or UPDRS-I at 12-months. Regarding scales assessing mood, only BDI-A subscores changed at 12-months (*P* = 0.047), but not the total BDI score. Furthermore, QUIP-RS scores improved at 6-months (*P* = 0.037) and at 12-months (median IQR change from baseline: −7.5 IQR 0.0–3.5; *P* = 0.008), but it was only significant after Bonferroni in the last one.

In the STN-DBS group, the NMSS total scores remained unchanged during the two assessment periods. However, there was a decrease in UPDRS-I scores at 6-months (*P* = <0.001) and 12-months (median IQR change from baseline: −4.5 IQR 3.0–2.5; *P* = <0.001), both significant after Bonferroni correction. Furthermore, at 6-months, HADS total scores (*P* = 0.005) and HADS-A subscores (*P* = 0.012) and HADS-D subscores (*P* = 0.016) lessened, and that were significant after Bonferroni correction. At 12-months total HADS (median IQR change from baseline: −6.5 IQR 5.7–6.0; *P* = <0.0001) and anxiety (*P* = <0.0001) and depression (*P* = 0.049) subscores were also reduced; however, the depression subscores was not significant after Bonferroni correction. In addition, BDI scores improved at 6 (*P* = 0.033) and 12-months (median IQR change from baseline: −2.0 IQR 4.7–6.0; *P* = <0.001), both significant after Bonferroni correction. Moreover, QUIP-RS scores were reduced at 6-months (*P* = 0.046) and at 12-months (median IQR change from baseline: −4.5 IQR 0–4.5; *P* = 0.012), but only the last one survived after Bonferroni correction. The ELFT score slightly worsened at 6-months (*P* = 0.010), and this persisted 12-months (median IQR change from baseline: −6.0 IQR 9.0–0.0; *P* = 0.001), both significant after multiple comparison. The only group that showed changes in CBI-R was LCIG (*P* = 0.012), but this was only seen at 6-months, including after the Bonferroni correction.

### Effect of DATs on motor fluctuations

At 6 and12-months the APO group had no reduction in the On-UPDRS-III, FOG-Q or UDysRS scores.

In the LCIG group, there were improvements in UPDRS-IV (*P* = 0.011), FOG-Q (*P* = 0.010) and UDysRS (*P* = 0.016) at 6-months, and these changes persisted after Bonferroni correction. However, at 12-months there was a reduction in UPDRS-IV (median IQR change from baseline: −4.0 IQR 4.3–3.5; *P* = 0.001) and UDysRS (median IQR change from baseline: −6.5 IQR 12.5–19.0; *P* = 0.026), although only the former survived after Bonferroni correction.

Additionally, FOG-Q scores were improved meaningfully at the 12-month assessment (median IQR change from baseline: −9.5 IQR 0–7.0; *P* = 0.004), including after multiple comparison analysis.

In the STN-DBS group, at 6-months, there was an improvement in UPDRS-II (*P* = 0.003), UPDRS-IV (*P* = <0.001) and UDysRS (*P* = <0.001), including after Bonferroni correction. The FOG-Q scores also improved (*P* = 0.034), but this was no significant after multiple comparison analysis. At 12-months, the UPDRS-II (median IQR change from baseline: −7.5 IQR 12.0–6.0; *P* = <0.001), UPDRS-IV (median IQR change from baseline: −6.0 IQR 6.0–7.0; *P* = <0.001), UDysRS (median IQR change from baseline: −23.0 IQR 0.0–30.0; *P* = <0.001) were improved.

In any of the three groups, SEADL scores were not changed at any of the two follow-up assessments.

### Effect of DATs on caregiver stress and burden

At 6-months, none of the three groups had a change in caregiver scales. At 12-months, only the STN-DBS had a reduction of ZCB (median IQR change from baseline: 7.5 IQR 0.0–7.0; *P* = 0.004) and CSI (median IQR change from baseline: 3.5 IQR 0.0–2.7; *P* = 0.004), including after Bonferroni correction.

## Discussion

In this prospective, single-centre real-life study of 66 patients with PD, we observed the effects of three different device-assisted therapies on quality of life, non-motor symptoms and motor fluctuations at 6-months and 12-months. Our study was designed to thoroughly assess most of the aspects of motor and non-motor symptoms in patients living with PD, and thus differs from previous comparative studies where clinimetric assessments were largely performed with a subset of scales (UPDRS-MDS, PDQ-8, NMSS). This allows us to compare and extend our understanding of the potential effects of every DAT, for instance, on freezing of gait, impulse control disorders and other neuropsychiatric aspects such as anxiety and depression. Furthermore, because our study design was pragmatic and non-randomised, the resulting cohorts were a reflection of real-life patient and clinician choices in treatment modality and outcomes. In PD patients, the severity and combination of motor and non-motor symptoms requiring treatment are expected to differ and may influence treatment choice.

Despite no systematic or intentional stratification of treatment recommendations according to pre-determined guidelines, there were several differences between groups at baseline that were observed. First, the STN-DBS group had better cognitive, non-motor and motor profiles than the LCIG and APO groups. Second, the LCIG had higher non-motor symptoms and longer disease duration compared to the other two groups. This may presumably reflect the differences in our perceptions of indications and contraindications for each therapy. Despite differences in disease duration and severity, the actual age at intervention and severity of motor complications, including dyskinesia, were not different between groups. Overall, our cohort is similar in age to other published cohorts comparing DATs^5,12,14^, including the Euroinf2 study^11^. In other similar studies, the STN-DBS group had lower NMSS baseline scores when compared to APO and LCIG-treated groups^11^. Our STN-DBS group also had better cognitive status, less depressive symptoms, and less severe freezing of gait scores compared to the APO and LCIG groups. These baseline differences need to be considered when comparing outcomes between groups.

In this study, we did not have any prespecified primary or secondary outcomes, but instead, we compared outcomes at two points from baseline with the aim to explore different outcomes based on several motor, non-motor, cognitive and caregiver scales. However, our statistical analysis was strict and adjusted for multiple comparison, with the caveat of a relatively small number of patients in APO and LCIG groups. We cannot exclude that some comparisons were not statistically significant due to type 2 error.

In our study, we found that PDQ-39 scores were not modified in the APO group, which is not dissimilar to what has been reported in large studies. In the TOLEDO study, a 12-week trial apomorphine infusion did not demonstrate changes in PDQ-8 scores, yet there was a 2.5-h reduction in “off” time, nevertheless without change in UPDRS-III scores^7^. Recently, a retrospective study of 110 patients with continuous apomorphine infusion (~80% of patients with a 24-h infusion), showed that only 65% continued with the therapy at 2 years, and that motor fluctuations (items 36–39 of UPDRS-IV) improved 32.4% at 24-months during this 2-year period^15^. However, PDQ8 scores, total UPDRS-IV and dyskinesias subscores were not modified. In contrast, previous studies have reported positive changes in PDQ-8 scores in patients with APO at 6-months^5,11^, although this improvement may not be sustained at 12-months^16^. The long-term effect of APO in QoL could partially explain the discontinuation rate ranging from 16.7% at 16-months^16^ and up to 37% to 66% after 2–3 years of treatment^17,18^. In terms of the other groups in our study, the LCIG group had a 21% reduction at 6 and 12-months in PDQ-39 SI, similar to what has been observed (21–34%) in other studies^11^. Furthermore, the STN-DBS group had a reduction of 41% in PDQ-39 SI at 12-months, which is relatively higher to previous studies showing a 17–27% reduction in QoL scales^11^.

Previous studies have established that neuropsychiatric symptoms in PD may correlate with caregiver burden and stress^19,20^, whereas the effect of patients’ motor disability on caregiver stress may be variable^21^. Our LCIG group did not show changes in caregiver burden despite the reduction of motor complications in LCIG. Nevertheless, an unexpected finding was the lessening of the Cambridge Behavioural Inventory (CBI-R) scores at 6-months with LCIG. This is a proxy scale that captures cognitive and behavioural symptoms as well as activities of daily living (ADL), with higher scores representing more severe symptoms. To date, there are no studies evaluating patients with LCIG and the effects on CBI-R, but our findings suggest that levodopa infusion does not impose any negative effects on behaviour or cognition in PD. In terms of the caregiver scales, conflicting data exist regarding LCIG therapy, with some reports of improvement in caregiver stress and burden^22^ and some not showing change after two years of follow-up^23^. In a recent study, despite LCIG improving PDQ-39, non-motor and motor scores in 59 patients with PD, there was no change in the QoL and burden scores in caregivers^24^. In our study, we acknowledge that cultural and local health service differences, and caregiver resilience may have influenced the lack of change in burden scores in the LCIG. Relatively good QoL and moderately low strain have been observed in caregiver studies of the Australian PD population^25^.

In our study, caregiver stress and burden was reduced at 12-months only in the STN-DBS group, though this group had less non-motor complications at baseline. In a recent systematic review of caregiver satisfaction with DBS for PD, better outcomes were seen in younger patients, with minor psychiatric complications and lower medication requirements^26^. Furthermore, it is possible that other several variables may have led to these changes. For instance, our STN-DBS patients had fewer non-motor symptoms and better cognitive scores, and if we consider that DBS requires less special maintenance after implantation, it is possible that this group could have become more independent from carers, thus resulting in lower caregiver scores at 12-months.

With a focus on non-motor symptoms, we did not detect any changes in NMSS, and UPDRS-I scales in the LCIG group. The positive effects of LCIG on non-motor symptoms at one and over a year have been established in previous large studies. It is of interest to note that in both the 12-month open-label and GLORIA studies^6,27^, mean improvements in NMSS and QoL peaked at 12-months rather than earlier. Furthermore, large LCIG cohort studies (DUOGLOBE) have reported a 26% improvement in NMSS scores at 12-months^28^.

In our study, despite clear differences in total NMSS scores at 12-months (40% reduction of total score), these changes were not significant; thus, it is possible that our strict statistical analysis and the relatively low number may have contributed to lack of statistical significance. In terms of the other group, we did not identify NMSS improvements with STN-DBS, which is in contrast to what other studies have reported^5,11^. Nonetheless, other measures of non-motor symptoms did actually improve in the STN-DBS group, including UPDRS-I at both 6 and 12-months. This can be partially explained at least by the lower burden of non-motor symptoms, younger age and shorter disease duration, which are variables known to contribute to non-motor symptoms burden.

Our results identified changes in mood scales after device-assisted therapies. At 6 and 12-months, the STN-DBS group improved depression and anxiety scores. Other STN-DBS studies have reported improvement in anxiety and depression at 6-months, which seems to correlate with the location of active contact location with more ventral STN stimulation^29^. In our centre, we tend to initiate DBS programming using a contact in the dorsal STN, which is generally the most efficacious stimulation site for control of motor symptoms^30^; however, we did not perform lead location analysis to see of this correlated with the improvement of depression and anxiety. Another finding in the STN-DBS group was a slight but significant reduction in ELFT at 6 and 12-months. This is in line with previous studies showing a mild decline in verbal fluency after STN-DBS^31,32^. Regarding the LCIG group, there was a reduction in anxiety scores at 12-months. In this regard, only one small cohort study has prospectively evaluated BDI-II scales in patients with LCIG, showing a significant decrease in depression scores (but not anxiety) after 6-months of therapy^33^. Although this suggests that LCIG may reduce anxiety and depression, this needs confirmation.

Our study is the first to prospectively compare DATs and changes in impulse control disorders (ICD). In our study, we did not find any significant effect of APO on the QUIP-RS scores. Some studies observed a low incidence of ICD with APO infusion, with a tendency for individuals to reduce previous behaviours, but long-term studies have not been reported^34^. In the 12-week and subsequent 64-week TOLEDO study, de novo ICDs were not observed^16^, but this may relate to the relatively short duration of treatment and follow-up. In our LCIG group, QUIP-RS scores were reduced substantially at 12-months, despite a considerable increase in LEDD. Overall, our data support previous findings that current DATs are effective in reducing QUIP-RS scores in most, but not necessarily all patients^35,36^. There are scarce data on the effect of LCIG in ICD symptoms, with some studies reporting a 64% improvement in QUIP-RS scores 6-months after treatment^5,37,38^. In the case of STN-DBS^39,40^, some studies reported an ICD remission rate of 60–69% in patients with an average 3-year follow-up. In our study, we identified a significant reduction in QUIP-RS scores only at 12-months with STN-DBS. Note that the QUIP-RS scores were lower at baseline in the STN-DBS group, potentially reflecting our practice of slowing dopamine agonists prior to surgery when addictive behaviours are identified.

In our analysis, we did not find a significant statistical difference in motor function scales in the APO group; however, there was a clinically important difference in UDysRS scores at 12-months according to comparative data^41,42^, which suggests that this group was underpowered by the small number of patients.

Our study consolidates previous observations on the anti-dyskinetic effects of LCIG infusion and STN-DBS in PD. We observed that both treatment modalities reduced motor complication severity and dyskinesias at 12-months. In contrast to other reports, our LCIG group increased the LEDD by 50% at 12-months, despite a 33% reduction of UPDRS-IV scores at 12-months. Dyskinesia scores (UDysRS) were reduced at 6-months (22%), and these changes persisted at 12-months; however, multiple comparison analysis was only significant at 6-months. This finding supports both a pharmacokinetic and pharmacodynamic effect of levodopa-carbidopa infusion on dyskinesia severity and duration^43,44^. The STN-DBS group had a 63% reduction in UPDRS-IV and 88% in UDysRS scores at 12-months and also a 58% reduction of the total LEDD, an observation also made in other studies^11,14^.

The severity of FOG was also improved by LCIG at 12-months but not with the other two therapies. In the LCIG group, there was a 50% reduction of FOG-Q scores, in keeping with other reports of its efficacy in both the OFF and ON periods FOG^45–48^. In the STN-DBS group, while using conventional stimulation parameters, there was an improvement of FOG severity at 6-months; however, this effect was no longer significant at 12-months. Several explanations may account for this finding, with one plausibly being a 50% reduction in total LEDD. Furthermore, it has been previously shown that STN-DBS can improve FOG compared to the “off-state”, especially with active electrodes within the STN, but does not have an additional effect to that provided by the best levodopa response^49^. Additional factors may include disease progression, progressive atrophy of the putamen^50^, and an adverse effect of the STN stimulation itself^51^.

The design of our study has some inherent limitations. The number of patients treated with APO was small compared to the LCIG and STN-DBS groups, probably resulting in insufficient power to detect significant changes in motor and non-motor scores. The lack of blinded assessments in our study is an additional limitation. The lack of randomisation could be considered a weakness of our study; however, our patients chose therapy based on preference as part of routine clinical practice after a detailed and balanced discussion of their treatment options. Therefore, we believe our study, even though non-randomised, will help to inform clinical practice.

Our study findings highlight the baseline differences of a cohort treated with the three different device-assisted therapies, which were a post-hoc finding from our analysis and not the result of explicit, pre-determined selection criteria. In our institution, patients can choose any of the three therapies, which are provided by the public health system without involving cost for them. Therapy selection was therefore determined by patients’ preferences and supported by their caregivers and treating neurologists, but not economic factors. However, subconscious bias can also influence treatment selection, and by its nature, will not be evident to the treating clinician.

Differences in baseline characteristics will likely have a significant influence on treatment outcomes, but without systematic review of their own patient cohorts, clinicians may ascribe differences in outcome solely to the effects of therapy, leading to inaccurate assumptions about which therapy may or may not be optimally recommended for future patients. We suggest that a systematic audit of baseline characteristics of patients, not just treatment outcomes, should be considered in all movement disorder services offering device-assisted therapies to better understand treatment practices and that baseline characteristics should be the starting point of any discussion comparing these therapies. While our results are similar to those reported by the Euroinf2 study, the multi-centre design of that study prevents conclusions about whether differences in baseline characteristics were driven more by centre or therapeutic choice. Any future prospective studies comparing DATs should ideally match or control for baseline patient characteristics in sufficient number to make equable comparisons between therapies.

## Methods

We performed a prospective, observational “real life” study of consecutive people with PD undergoing DAT. Patients were enrolled from 2014 to 2019 in the Movement Disorders Unit, Neurology Department at Westmead Hospital. This study was approved by the Western Sydney Local Health District Human Research and Ethics Committee (HREC2013/3/6.2 [3674]). All participants provided written informed consent to take part in the study. The choice of DAT was made by the patient as per routine clinical care.

### Patient selection

We included consecutive patients proceeding with a DAT for PD (Fig.1). Exclusion criteria included an alternative clinical diagnosis, if the patient was unable to comply with the study requirements, unable to give informed consent, or declined participation. Treatment was not randomised, but instead decided between the patient and treating clinician according to routine clinical care, based on factors such as patient preference and suitability for each of the treatment options in the opinion of the treating movement disorder specialist. After interest in pursuing a DAT was established in the course of routine clinical follow-up, for therapy selection, the patient and his family caregiver were first brought to a session where the specialist nurses and neurologists discussed the inherent characteristics of each of the therapies (APO, LCIG or STN-DBS). In a different appointment, the patient then made the choice of a particular therapy based on those previous discussions.

All patients included in the study were seen at least twice in our Movement Disorders Clinic before study enrolment. As part of our standard clinical care, patients were examined to confirm the diagnosis of PD, based on clinical history, examination and supportive tests such as neuroimaging to exclude secondary Parkinsonism. Levodopa responsiveness was first explored by history, however all patients had a formal levodopa challenge in their practically defined “off-state”, where the nature of motor fluctuations, including FOG were evaluated. Current dopaminergic medication, and other comorbidities were also considered. There were no patients with clinical neuropathy enrolled on our study. Patients that opted for STN-DBS underwent neuropsychological and psychiatric assessments at baseline to evaluate possible untreated mood disorders.

### Clinimetric assessments at baseline and 6 and 12 months

Demographic and clinical data of the patients and their caregivers were collected at baseline, 6-months and 12-months.

We collected the following data at each study visit:

i. Levodopa equivalent daily dose (LEDD)^52^
ii. Quality of life: 39 items Parkinson’s disease questionnaire (PDQ-39) summary index (SI)^53,54^
iii. Motor function: Hoehn & Yahr Scale (HY), the MDS-Unified Parkinson’s Disease Rating Scale (UPDRS-III), UPDRS-II^55^, Schwab and England Activities of Daily Living Scale (SEADL), UPDRS-IV, Unified Dyskinesia Rating Scale (UDysRS)^56,57^, New Freezing of Gait Questionnaire (FOG-Q)^58^. It should be noted that only the practically defined, medicated ON-state UPDRS-III motor scores were used for pre-and post-treatment comparisons, not OFF scores. Motor diaries were found to be unreliable in our culturally, linguistically and educationally diverse population.
iv. Non-motor function: UPDRS-I, Non-Motor Symptoms Scale (NMSS)^59^, Beck Depression Inventory (BDI)^60^, Hospital Anxiety and Depression Scale (HADS)^61^, Questionnaire for Impulsive-compulsive Control Disorders in Parkinson’s Disease (QUIP-RS)^62^, Montreal Cognitive Assessment (MOCA)^63^, Excluded Letter Fluency test (ELFT)^64^, and Addenbrooke’s Cognitive Examination III (ACE-III)^65^, Cambridge Behavioural Inventory Revised (CBI-R)^66^.
v. Caregiver scales: Zarit Caregiver Burden (ZCB)^67^, Caregiver Strain Index (CSI)^22^.

### Statistical analysis

We first assessed for data distribution using the Shapiro-Wilk test. All data except for age, LEDD and UPDRS-I were not normally distributed. Therefore, to compare baseline differences in the three groups, we performed a Kruskal–Wallis test for between-group comparisons. To compare the change of scores within subjects at baseline, 6-months and 12-months, we applied the Friedman test. For variables with statistically significant differences on the Friedman test, we then performed a post-hoc Kruskal–Wallis test with Bonferroni correction to analyse differences between baseline and 6-month and baseline and 12-months. Data for all analyses is expressed in the median and interquartile range (IQR). Values of *p* < 0.05 were set as statistically significant (including, when performed, after Bonferroni correction). If significant at 12-months, the median with interquartile (IQR) change from baseline was calculated for PDQ-39 SI, NMSS, UPDRS-I, UPDRS-II, UPDRS-IV, UDysRS and FOG-Q. We used SPSS software version 23 for statistical analysis.

### Reporting summary

Further information on research design is available in the Nature Research Reporting Summary linked to this article.

## Supplementary information

Supplemental material

reporting summary

## Data Availability

The data that support the findings of this study are available from the corresponding author.
