# Supplementary material for: A single centre prospective study of three device-assisted therapies for Parkinson’s disease
Source: NPJ Parkinsons Dis. 2023 Jun 29;9:101. doi: 10.1038/s41531-023-00525-w (PMC10310730; doi:10.1038/s41531-023-00525-w)
Supplement: Supplementary file 1 — Supplemental material [file 41531_2023_525_MOESM1_ESM.pdf]

Supplementary material

Adverse drug event or device related events during the 12-months follow-up

	APO	STN-DBS	LCIG	Total cohort (n=63)
**Patients with at least one side effect**	11	20	9	44
Deaths	0	0	0	0
Chronic problematic orthostatic hypotension or dizziness	0	0	0	0
New onset polyneuropathy	0	0	0	0
Weight loss	0	0	0	0
Chronic abdominal pain	0	0	0	0
**Non-motor related side effects**				
Hallucinations	1	1	1	3
Depression	0	1	0	1
Apathy	0	3	0	3
Impulse control disorder	3	0	1	4
Insomnia	1	0	0	1
Increase in RBD	0	2	0	2
Excessive daytime somnolence	2	0	0	2
Anxiety	1	1	0	2
Cognitive change	1	0	2	3
Dopamine agonist withdrawal syndrome	1	0	0	1
Headache	0	1	0	1
**Motor related side effects**				
Troublesome dyskinesia	1	0	0	1
Non-troublesome dyskinesia	0	1	2	3
Falls	0	1	0	1
Gait problem	0	1	0	1
Speech problems	0	4	0	4
Freezing of gait	2	3	1	6
Exacerbation of Parkinsonism	2	0	0	2
**Device related**				
Complication of device insertion	0	0	0	0
Device related infection	0	0	0	0
Postoperative wound infection	0	0	0	0
Device dislocation	0	0	0	0
Device occlusion	0	0	0	0
Device issue	0	1	0	1
Excessive granulation tissue	0	0	2	3
Local skin reaction	0	0	0	0

STN-DBS stimulation parameters at 12-months

ID	
001	L STN 1-/C+ 2.9v/60us/130Hz/1517Ω/1.9mA<br>R STN 10-/C+ 3.2v/60us/130Hz/1252Ω/2.5mA
002	L STN 2-/C+ 2.3v/120us/70Hz/1513Ω/1.5mA<br>R STN 10-/C+ 2.3v/120us/70Hz/1209Ω/1.9mA
004	L STN 1-/C+ 2.5v/60us/130Hz/1355Ω/1.9mA<br>R STN 9-/C+ 2.0v/60us/130Hz/1375Ω/1.5mA
014	L STN 2-/C+ 3.7v/60us/130Hz/975Ω/3.8mA<br>R STN 10-/C+ 3.5v/60us/130Hz/981Ω/3.5mA
015	L STN 1-/C+ 4.5v/120us/70Hz/1149Ω/3.9mA<br>R STN 9-/C+ 2.5v/90us/70Hz/1237Ω/2.0mA
020	L STN 1-/C+ 2.9v/60us/130Hz/1307Ω/2.2mA<br>R STN 10-/C+ 2.9v/60us/130Hz/1134Ω/2.6mA
028	L STN 1-/C+ 2.7v/60us/130Hz/1255Ω/2.2mA<br>R STN 9-/C+ 4.3v/70us/130Hz/1262Ω/3.5mA
029	L STN 1-/C+ 3.2v/60us/130Hz/1174Ω/2.7mA<br>R STN 9-/C+ 3.1v/60us/130Hz/1328Ω/2.4mA
033	L STN 1-/C+ 4.0v/60us/130Hz/1089Ω/3.7mA<br>R STN 9-/C+ 0.7v/60us/130Hz/1584Ω/0.4mA
040	L STN 2-/C+ 2.3v/60us/130Hz/1518Ω/1.5mA<br>R STN 10-/C+ 3.1v/60us/130Hz/1123Ω/2.8mA
046	L STN 1-/C+ 4.1v/60us/130Hz/1134Ω/3.7mA<br>R STN 9-/C+ 0.0v/60us/130Hz/----Ω/---mA
047	L STN 2-/C+ 1.1v/60us/130Hz/1775Ω/0.6mA<br>R STN 10-/C+ 2.6v/60us/130Hz/1212Ω/2.2mA
049	L STN 0-/C+ 2.8v/60us/130Hz/1186Ω/2.4mA<br>R STN 8-/C+ 3.3v/60us/130Hz/1144Ω/2.9mA
050	L STN 1-/C+ 2.1v/60us/130Hz/1314Ω/1.6mA<br>R STN 10-/C+ 2.3v/60us/130Hz/1317Ω/1.7mA
056	L STN 1 0-/C+ 2.90v/60us/125Hz/1111 Ω/2.6mA<br>L STN 2 3-/C+ 1.50v/60us/125Hz/1179 Ω/1.2mA<br>R STN 2 11-/C+ 1.50v/60us/125Hz/1102 Ω/1.3mA<br>R STN 1 9-/C+ 2.30v/60us/125Hz/1102 Ω/2.2mA
060	L STN 1-/C+ 2.7v/60us/130Hz/1037Ω/2.6mA<br>R STN 9-/C+ 3.4v/60us/130Hz/1042Ω/3.2mA
063	L STN 1-/C+ 2.9v/60us/130Hz/1200 Ω/2.423mA<br>R STN 9-/C+ 2.9v/60us/130Hz/1238 Ω/2.338mA
064	L STN 3-/C+ 3.0v/60us/130Hz/1081Ω/2.8mA<br>R STN 11-/C+ 3.2v/60us/130Hz/1267Ω/2.8mA
066	L STN 3-/C+ 2.90v/60us/130Hz/274Ω/3.3mA<br>R STN 10-/C+ 2.70v/60us/130Hz/976Ω/2.8mA
070	L STN 1-/C+ 1.90v/60us/130Hz/1213 Ω/1.5mA<br>R STN 8-/C+ 1.50v/60us/130Hz/1060 Ω/1.4mA
071	L STN 2-/C+ 2.15v/60us/130Hz/1424 Ω/1.5mA

	R STN 10-/c+ 2.15v/60us/130Hz/1138 Ω /1.9mA
072	L STN 2-/C+ 3.2v/60us/130Hz/969 Ω/3.3mA<br>R STN 10-/C+ 3.0v/60us/130Hz/1006 Ω/3.0mA
075	L STN 1-/C+ 2.5v/60us/130Hz/982Ω/2.6mA<br>R STN 9-/C+ 2.5v/60us/130Hz/1391Ω/1.8mA
076	L STN 1-/C+ 2.95/60us/130Hz/1445 Ω/2.0mA<br>R STN 9-/C+ 2.15/60us/130Hz/1053 Ω/2.0mA
077	L STN 1-/C+ 1.9v/60us/130Hz/1325Ω/1.4mA<br>R STN 9-/C+ 2.2v/60us/130Hz/1285Ω/1.7mA
078	L STN 0-/1-/C+ 2.9v/70us/130Hz/1288 Ω/2.2mA<br>R STN 9-/C+ 4.0v/90us/130Hz/1166 Ω/3.4mA
079	L STN 1-/C+ 1.65v/60us/130Hz/1197 Ω/1.4mA<br>R STN 9-/C+ 2.35v/60us/130Hz/1296 Ω/1.8mA
080	L STN 1-/C+ 2.45v/60us/130Hz/1306Ω/1.9mA<br>R STN 10-/C+ 2.65v/60us/130Hz/1067Ω/2.5mA
082	L STN 1-/C+ 2.80v/60us/130Hz/1549 Ω/1.8mA<br>R STN 9-/C+ 2.80v/60us/130Hz/1565 Ω/1.7mA
083	L STN 1-/C+ 3.3v/60us/130Hz/1521 Ω/2.2mA<br>R STN 9-/C+ 3.3v/60us/130Hz/1236 Ω/2.7mA
084	L STN 1-/C+ 2.35v/60us/130hz/1086 Ω/2.1mA<br>R STN 9-/C+ 2.35v/60us/130hz/1086 Ω/1.6mA
008	L STN 2-/C+ 2.6v/60us/130Hz/1326 Ω/1.9mA<br>R STN 10-/C+ 2.6v/60us/130Hz/1193 Ω/2.2mA
---	L STN 0-/C+ 3.4v/60us/130Hz/1202 Ω/2.8mA<br>R STN 8-/C+ 2.9v/60us/130Hz/1083 Ω/2.7mA
036	L STN 1-/C+ 2.5v/60us/130Hz/1295 Ω/1.9mA<br>R STN 10-/C+ 2.8v/60us/130Hz/1388 Ω/1.9mA
