# Supplementary material for: A single centre prospective study of three device-assisted therapies for Parkinson’s disease
Source: NPJ Parkinsons Dis. 2023 Jun 29;9:101. doi: 10.1038/s41531-023-00525-w (PMC10310730; doi:10.1038/s41531-023-00525-w)
Supplement: Supplementary file 2 — reporting summary [file 41531_2023_525_MOESM2_ESM.pdf]

nature portfolio

Corresponding author(s): Fung SC Victor. et al. NPJPARKD-01667R2

Double-blind peer review submissions: write DBPR and your manuscript number here instead of author names.

Last updated by author(s): 2023-04-25

Reporting Summary

Nature Portfolio wishes to improve the reproducibility of the work that we publish. This form provides structure for consistency and transparency in reporting. For further information on Nature Portfolio policies, see our Editorial Policies and the Editorial Policy Checklist.

Statistics

For all statistical analyses, confirm that the following items are present in the figure legend, table legend, main text, or Methods section.

n/a	Confirmed	
☐	☑	The exact sample size (n) for each experimental group/condition, given as a discrete number and unit of measurement
☐	☑	A statement on whether measurements were taken from distinct samples or whether the same sample was measured repeatedly
☐	☑	The statistical test(s) used AND whether they are one- or two-sided<br>*Only common tests should be described solely by name; describe more complex techniques in the Methods section.*
☐	☑	A description of all covariates tested
☐	☑	A description of any assumptions or corrections, such as tests of normality and adjustment for multiple comparisons
☐	☑	A full description of the statistical parameters including central tendency (e.g. means) or other basic estimates (e.g. regression coefficient) AND variation (e.g. standard deviation) or associated estimates of uncertainty (e.g. confidence intervals)
☐	☑	For null hypothesis testing, the test statistic (e.g. F, t, r) with confidence intervals, effect sizes, degrees of freedom and P value noted<br>*Give P values as exact values whenever suitable.*
☒	☐	For Bayesian analysis, information on the choice of priors and Markov chain Monte Carlo settings
☒	☐	For hierarchical and complex designs, identification of the appropriate level for tests and full reporting of outcomes
☒	☐	Estimates of effect sizes (e.g. Cohen's d, Pearson's r), indicating how they were calculated

Our web collection on statistics for biologists contains articles on many of the points above.

Software and code

Policy information about availability of computer code

Data collection	*Provide a description of all commercial, open source and custom code used to collect the data in this study, specifying the version used OR state that no software was used.*<br>excel windows
Data analysis	*Provide a description of all commercial, open source and custom code used to analyse the data in this study, specifying the version used OR state that no software was used.*<br>spss23

For manuscripts utilizing custom algorithms or software that are central to the research but not yet described in published literature, software must be made available to editors and reviewers. We strongly encourage code deposition in a community repository (e.g. GitHub). See the Nature Portfolio guidelines for submitting code & software for further information.

Data

Policy information about availability of data

All manuscripts must include a data availability statement. This statement should provide the following information, where applicable:

- Accession codes, unique identifiers, or web links for publicly available datasets
- A description of any restrictions on data availability
- For clinical datasets or third party data, please ensure that the statement adheres to our policy

Provide your data availability statement here.

The data that supports the findings of this study is available from the corresponding author.

Human research participants

Policy information about studies involving human research participants and Sex and Gender in Research.

Reporting on sex and gender

Use the terms sex (biological attribute) and gender (shaped by social and cultural circumstances) carefully in order to avoid confusing both terms. Indicate if findings apply to only one sex or gender; describe whether sex and gender were considered in study design whether sex and/or gender was determined based on self-reporting or assigned and methods used. Provide in the source data disaggregated sex and gender data where this information has been collected, and consent has been obtained for sharing of individual-level data; provide overall numbers in this Reporting Summary. Please state if this information has not been collected. Report sex- and gender-based analyses where performed, justify reasons for lack of sex- and gender-based analysis.

The number of male and female patients are described in the text

Population characteristics

Describe the covariate-relevant population characteristics of the human research participants (e.g. age, genotypic information, past and current diagnosis and treatment categories). If you filled out the behavioural & social sciences study design questions and have nothing to add here, write "See above."

Patients with clinical diagnosis of Parkinson's disease

Recruitment

Describe how participants were recruited. Outline any potential self-selection bias or other biases that may be present and how these are likely to impact results.

Consecutive recruitment of patients referred to our center for treatment with device-assisted therapies.

Ethics oversight

Identify the organization(s) that approved the study protocol.

Western Sydney Local Health district Human Research and Ethics Committee
protocol number: HREC2013/3/6.2 (3674)

Note that full information on the approval of the study protocol must also be provided in the manuscript.

Field-specific reporting

Please select the one below that is the best fit for your research. If you are not sure, read the appropriate sections before making your selection.

- [x] Life sciences
- [ ] Behavioural & social sciences
- [ ] Ecological, evolutionary & environmental sciences

For a reference copy of the document with all sections, see nature.com/documents/nr-reporting-summary-flat.pdf

Life sciences study design

All studies must disclose on these points even when the disclosure is negative.

Sample size

Describe how sample size was determined, detailing any statistical methods used to predetermine sample size OR if no sample-size calculation was performed, describe how sample sizes were chosen and provide a rationale for why these sample sizes are sufficient.

Our study did not require sample size calculation as it was observational.

Data exclusions

Describe any data exclusions. If no data were excluded from the analyses, state so OR if data were excluded, describe the exclusions and the rationale behind them, indicating whether exclusion criteria were pre-established.

No data were excluded for the analyses

Replication

Describe the measures taken to verify the reproducibility of the experimental findings. If all attempts at replication were successful, confirm this OR if there are any findings that were not replicated or cannot be reproduced, note this and describe why.

Our statistical analysis was performed by two different researchers using the same method

Randomization

Describe how samples/organisms/participants were allocated into experimental groups. If allocation was not random, describe how covariates were controlled OR if this is not relevant to your study, explain why.

Our study sis not involved randomization of treatment as our design was observational real-life study.

Blinding

Describe whether the investigators were blinded to group allocation during data collection and/or analysis. If blinding was not possible, describe why OR explain why blinding was not relevant to your study.

Our study was not blinded

Behavioural & social sciences study design

All studies must disclose on these points even when the disclosure is negative.

Study description

Briefly describe the study type including whether data are quantitative, qualitative, or mixed-methods (e.g. qualitative cross-sectional, quantitative experimental, mixed-methods case study).

NOT APPLICABLE

Research sample

State the research sample (e.g. Harvard university undergraduates, villagers in rural India) and provide relevant demographic information (e.g. age, sex) and indicate whether the sample is representative. Provide a rationale for the study sample chosen. For studies involving existing datasets, please describe the dataset and source.

NOT APPLICABLE

Sampling strategy

Describe the sampling procedure (e.g. random, snowball, stratified, convenience). Describe the statistical methods that were used to predetermine sample size OR if no sample-size calculation was performed, describe how sample sizes were chosen and provide a rationale for why these sample sizes are sufficient. For qualitative data, please indicate whether data saturation was considered, and what criteria were used to decide that no further sampling was needed.

NOT APPLICABLE

Data collection

Provide details about the data collection procedure, including the instruments or devices used to record the data (e.g. pen and paper, computer, eye tracker, video or audio equipment) whether anyone was present besides the participant(s) and the researcher, and whether the researcher was blind to experimental condition and/or the study hypothesis during data collection.

NOT APPLICABLE

Timing

Indicate the start and stop dates of data collection. If there is a gap between collection periods, state the dates for each sample cohort.

NOT APPLICABLE

Data exclusions

If no data were excluded from the analyses, state so OR if data were excluded, provide the exact number of exclusions and the rationale behind them, indicating whether exclusion criteria were pre-established.

NOT APPLICABLE

Non-participation

State how many participants dropped out/declined participation and the reason(s) given OR provide response rate OR state that no participants dropped out/declined participation.

NOT APPLICABLE

Randomization

If participants were not allocated into experimental groups, state so OR describe how participants were allocated to groups, and if allocation was not random, describe how covariates were controlled.

NOT APPLICABLE

Ecological, evolutionary & environmental sciences study design

All studies must disclose on these points even when the disclosure is negative.

Study description

Briefly describe the study. For quantitative data include treatment factors and interactions, design structure (e.g. factorial, nested, hierarchical), nature and number of experimental units and replicates.

NOT APPLICABLE

Research sample

Describe the research sample (e.g. a group of tagged Passer domesticus, all Stenocereus thurberi within Organ Pipe Cactus National Monument), and provide a rationale for the sample choice. When relevant, describe the organism taxa, source, sex, age range and any manipulations. State what population the sample is meant to represent when applicable. For studies involving existing datasets, describe the data and its source.

NOT APPLICABLE

Sampling strategy

Note the sampling procedure. Describe the statistical methods that were used to predetermine sample size OR if no sample-size calculation was performed, describe how sample sizes were chosen and provide a rationale for why these sample sizes are sufficient.

NOT APPLICABLE

Data collection

Describe the data collection procedure, including who recorded the data and how.

Timing and spatial scale

Indicate the start and stop dates of data collection, noting the frequency and periodicity of sampling and providing a rationale for these choices. If there is a gap between collection periods, state the dates for each sample cohort. Specify the spatial scale from which the data are taken

NOT APPLICABLE

Data exclusions

If no data were excluded from the analyses, state so OR if data were excluded, describe the exclusions and the rationale behind them, indicating whether exclusion criteria were pre-established.

NOT APPLICABLE

Reproducibility

Describe the measures taken to verify the reproducibility of experimental findings. For each experiment, note whether any attempts to repeat the experiment failed OR state that all attempts to repeat the experiment were successful.

NOT APPLICABLE

Randomization

Describe how samples/organisms/participants were allocated into groups. If allocation was not random, describe how covariates were controlled. If this is not relevant to your study, explain why.

NOT APPLICABLE

Blinding

Describe the extent of blinding used during data acquisition and analysis. If blinding was not possible, describe why OR explain why blinding was not relevant to your study.

NOT APPLICABLE

Did the study involve field work? ☐ Yes ☐ No

Field work, collection and transport

Field conditions

Describe the study conditions for field work, providing relevant parameters (e.g. temperature, rainfall).

NOT APPLICABLE

Location

State the location of the sampling or experiment, providing relevant parameters (e.g. latitude and longitude, elevation, water depth).

NOT APPLICABLE

Access & import/export

Describe the efforts you have made to access habitats and to collect and import/export your samples in a responsible manner and in compliance with local, national and international laws, noting any permits that were obtained (give the name of the issuing authority, the date of issue, and any identifying information).

NOT APPLICABLE

Disturbance

Describe any disturbance caused by the study and how it was minimized.

NOT APPLICABLE

Reporting for specific materials, systems and methods

We require information from authors about some types of materials, experimental systems and methods used in many studies. Here, indicate whether each material, system or method listed is relevant to your study. If you are not sure if a list item applies to your research, read the appropriate section before selecting a response.

Materials & experimental systems

n/a	Involved in the study
☒	☐ Antibodies
☒	☐ Eukaryotic cell lines
☒	☐ Palaeontology and archaeology
☒	☐ Animals and other organisms
☒	☐ Clinical data
☒	☐ Dual use research of concern

Methods

n/a	Involved in the study
☒	☐ ChIP-seq
☒	☐ Flow cytometry
☒	☐ MRI-based neuroimaging

Antibodies

Antibodies used	*Describe all antibodies used in the study; as applicable, provide supplier name, catalog number, clone name, and lot number.* NOT APPLICABLE
Validation	*Describe the validation of each primary antibody for the species and application, noting any validation statements on the manufacturer's website, relevant citations, antibody profiles in online databases, or data provided in the manuscript.* NOT APPLICABLE

Eukaryotic cell lines

Policy information about cell lines and Sex and Gender in Research

Cell line source(s)	*State the source of each cell line used and the sex of all primary cell lines and cells derived from human participants or vertebrate models.* NOT APPLICABLE
Authentication	*Describe the authentication procedures for each cell line used OR declare that none of the cell lines used were authenticated.* NOT APPLICABLE
Mycoplasma contamination	*Confirm that all cell lines tested negative for mycoplasma contamination OR describe the results of the testing for mycoplasma contamination OR declare that the cell lines were not tested for mycoplasma contamination.* NOT APPLICABLE
Commonly misidentified lines (See ICLAC register)	*Name any commonly misidentified cell lines used in the study and provide a rationale for their use.* NOT APPLICABLE

Palaeontology and Archaeology

Specimen provenance	*Provide provenance information for specimens and describe permits that were obtained for the work (including the name of the issuing authority, the date of issue, and any identifying information). Permits should encompass collection and, where applicable, export.* NOT APPLICABLE
Specimen deposition	*Indicate where the specimens have been deposited to permit free access by other researchers.* NOT APPLICABLE
Dating methods	*If new dates are provided, describe how they were obtained (e.g. collection, storage, sample pretreatment and measurement), where they were obtained (i.e. lab name), the calibration program and the protocol for quality assurance OR state that no new dates are provided.* NOT APPLICABLE

☐ Tick this box to confirm that the raw and calibrated dates are available in the paper or in Supplementary Information.

Ethics oversight	*Identify the organization(s) that approved or provided guidance on the study protocol, OR state that no ethical approval or guidance was required and explain why not.* NOT APPLICABLE

Note that full information on the approval of the study protocol must also be provided in the manuscript.

Animals and other research organisms

Policy information about studies involving animals; ARRIVE guidelines recommended for reporting animal research, and Sex and Gender in Research

Laboratory animals	*For laboratory animals, report species, strain and age OR state that the study did not involve laboratory animals.* NOT APPLICABLE

Wild animals	*Provide details on animals observed in or captured in the field; report species and age where possible. Describe how animals were caught and transported and what happened to captive animals after the study (if killed, explain why and describe method; if released, say where and when) OR state that the study did not involve wild animals.*	NOT APPLICABLE
Reporting on sex	*Indicate if findings apply to only one sex; describe whether sex was considered in study design, methods used for assigning sex. Provide data disaggregated for sex where this information has been collected in the source data as appropriate; provide overall numbers in this Reporting Summary. Please state if this information has not been collected. Report sex-based analyses where performed, justify reasons for lack of sex-based analysis.*	NOT APPLICABLE
Field-collected samples	*For laboratory work with field-collected samples, describe all relevant parameters such as housing, maintenance, temperature, photoperiod and end-of-experiment protocol OR state that the study did not involve samples collected from the field.*	NOT APPLICABLE
Ethics oversight	*Identify the organization(s) that approved or provided guidance on the study protocol, OR state that no ethical approval or guidance was required and explain why not.*	NOT APPLICABLE

Note that full information on the approval of the study protocol must also be provided in the manuscript.

Clinical data

Policy information about clinical studies

All manuscripts should comply with the ICMJE guidelines for publication of clinical research and a completed CONSORT checklist must be included with all submissions.

Clinical trial registration	*Provide the trial registration number from ClinicalTrials.gov or an equivalent agency.*	Our study was not registered.
Study protocol	*Note where the full trial protocol can be accessed OR if not available, explain why.*	Study protocol can be provided upon request from other clinical researchers with expert in movement disorders..
Data collection	*Describe the settings and locales of data collection, noting the time periods of recruitment and data collection.*	Data was collected at baseline, 6- and 12-months points after treatment.
Outcomes	*Describe how you pre-defined primary and secondary outcome measures and how you assessed these measures.*	Our study did not specify clinical outcomes before treatment.

Dual use research of concern

Policy information about dual use research of concern

Hazards

Could the accidental, deliberate or reckless misuse of agents or technologies generated in the work, or the application of information presented in the manuscript, pose a threat to:

No	Yes	
[x]	[ ]	Public health
[x]	[ ]	National security
[x]	[ ]	Crops and/or livestock
[x]	[ ]	Ecosystems
[x]	[ ]	Any other significant area

Experiments of concern

Does the work involve any of these experiments of concern:

No	Yes	
[x]	[ ]	Demonstrate how to render a vaccine ineffective
[x]	[ ]	Confer resistance to therapeutically useful antibiotics or antiviral agents
[x]	[ ]	Enhance the virulence of a pathogen or render a nonpathogen virulent
[x]	[ ]	Increase transmissibility of a pathogen
[x]	[ ]	Alter the host range of a pathogen
[x]	[ ]	Enable evasion of diagnostic/detection modalities
[x]	[ ]	Enable the weaponization of a biological agent or toxin
[x]	[ ]	Any other potentially harmful combination of experiments and agents

ChIP-seq

Data deposition

☐ Confirm that both raw and final processed data have been deposited in a public database such as GEO.

☐ Confirm that you have deposited or provided access to graph files (e.g. BED files) for the called peaks.

Data access links<br>*May remain private before publication.*	NOT APPLICABLE
Files in database submission	NOT APPLICABLE
Genome browser session<br>(e.g. UCSC)	NOT APPLICABLE

Methodology

Replicates	NOT APPLICABLE
Sequencing depth	NOT APPLICABLE
Antibodies	NOT APPLICABLE
Peak calling parameters	NOT APPLICABLE
Data quality	NOT APPLICABLE
Software	NOT APPLICABLE

Flow Cytometry

Plots

NOT APPLICABLE

Confirm that:

☐ The axis labels state the marker and fluorochrome used (e.g. CD4-FITC).

☐ The axis scales are clearly visible. Include numbers along axes only for bottom left plot of group (a 'group' is an analysis of identical markers).

☐ All plots are contour plots with outliers or pseudocolor plots.

☐ A numerical value for number of cells or percentage (with statistics) is provided.

Methodology

Sample preparation	NOT APPLICABLE
Instrument	*Identify the instrument used for data collection, specifying make and model number.*
Software	*Describe the software used to collect and analyze the flow cytometry data. For custom code that has been deposited into a community repository, provide accession details.*
Cell population abundance	*Describe the abundance of the relevant cell populations within post-sort fractions, providing details on the purity of the samples and how it was determined.*
Gating strategy	*Describe the gating strategy used for all relevant experiments, specifying the preliminary FSC/SSC gates of the starting cell population, indicating where boundaries between "positive" and "negative" staining cell populations are defined.*

☐ Tick this box to confirm that a figure exemplifying the gating strategy is provided in the Supplementary Information.

Magnetic resonance imaging

Experimental design

Design type	NOT APPLICABLE

Design specifications

Specify the number of blocks, trials or experimental units per session and/or subject, and specify the length of each trial or block (if trials are blocked) and interval between trials.

NOT APPLICABLE

Behavioral performance measures

State number and/or type of variables recorded (e.g. correct button press, response time) and what statistics were used to establish that the subjects were performing the task as expected (e.g. mean, range, and/or standard deviation across subjects).

Acquisition

Imaging type(s)

Specify functional, structural, diffusion, perfusion.

NOT APPLICABLE

Field strength

Specify in Tesla

Sequence & imaging parameters

Specify the pulse sequence type (gradient echo, spin echo, etc.), imaging type (EPI, spiral, etc.), field of view, matrix size, slice thickness, orientation and TE/TR/flip angle.

Area of acquisition

State whether a whole brain scan was used OR define the area of acquisition, describing how the region was determined.

Diffusion MRI ☐ Used ☐ Not used

Preprocessing

Preprocessing software

Provide detail on software version and revision number and on specific parameters (model/functions, brain extraction, segmentation, smoothing kernel size, etc.).

NOT APPLICABLE

Normalization

If data were normalized/standardized, describe the approach(es): specify linear or non-linear and define image types used for transformation OR indicate that data were not normalized and explain rationale for lack of normalization.

Normalization template

Describe the template used for normalization/transformation, specifying subject space or group standardized space (e.g. original Talairach, MNI305, ICBM152) OR indicate that the data were not normalized.

Noise and artifact removal

Describe your procedure(s) for artifact and structured noise removal, specifying motion parameters, tissue signals and physiological signals (heart rate, respiration).

Volume censoring

Define your software and/or method and criteria for volume censoring, and state the extent of such censoring.

Statistical modeling & inference

Model type and settings

Specify type (mass univariate, multivariate, RSA, predictive, etc.) and describe essential details of the model at the first and second levels (e.g. fixed, random or mixed effects; drift or auto-correlation).

NOT APPLICABLE

Effect(s) tested

Define precise effect in terms of the task or stimulus conditions instead of psychological concepts and indicate whether ANOVA or factorial designs were used.

Specify type of analysis: ☐ Whole brain ☐ ROI-based ☐ Both

Statistic type for inference
(See Eklund et al. 2016)

Specify voxel-wise or cluster-wise and report all relevant parameters for cluster-wise methods.

Correction

Describe the type of correction and how it is obtained for multiple comparisons (e.g. FWE, FDR, permutation or Monte Carlo).

Models & analysis

n/a	Involved in the study
☐	☐ Functional and/or effective connectivity
☐	☐ Graph analysis
☐	☐ Multivariate modeling or predictive analysis

NOT APPLICABLE

Functional and/or effective connectivity

Report the measures of dependence used and the model details (e.g. Pearson correlation, partial correlation, mutual information).

Graph analysis

Report the dependent variable and connectivity measure, specifying weighted graph or binarized graph, subject- or group-level, and the global and/or node summaries used (e.g. clustering coefficient, efficiency, etc.).

Multivariate modeling and predictive analysis

Specify independent variables, features extraction and dimension reduction, model, training and evaluation metrics.
